# The Significance of Renal Impairment in Children with Eating Disorders

**DOI:** 10.3390/jpm15020056

**Published:** 2025-01-29

**Authors:** Avisha Meleika Hamilton, Michael Eisenhut

**Affiliations:** Paediatric Department, Luton & Dunstable University Hospital, Lewsey Road, Luton LU4 0DZ, UK

**Keywords:** dehydration, anorexia nervosa, percentage drop in creatinine, GFR, eating disorders

## Abstract

**Background:** Eating disorders have previously been associated with renal impairment. Low muscle mass reduces serum creatinine used for the calculation of the estimated glomerular filtration rate (eGFR), leading to overestimation of renal function. To solve this problem, the development of a tool to detect renal impairment in individual patients with a specific muscle mass is required to individualize risk assessment for further management. The objectives of our study were as follows: To investigate the percentage drop in creatinine (pdCr) during rehydration as a new indicator of renal dysfunction not dependent on muscle mass and to investigate a correlation between cardiovascular function and fluid management with renal function. **Methods**: In a 5-year retrospective cohort study of all consecutive children admitted because of an eating disorder, renal function expressed as eGFR on admission and as pdCr between admission and the lowest creatinine level was analysed in relation to cardiovascular parameters and fluid management. **Results:** We included 29 patients. The mean age was 13.4 years. A pdCr after admission was noted in 26/29 (89.7%). The eGFR was <90 in 15 (65%) and improved to >90 in 13/15 (86.6%). In patients with a fluid management plan, there was a median of 18.6% for those with pdCr and 6.4% (*p* = 0.02) for those without. Renal dysfunction was not related to cardiovascular parameters. **Conclusions:** The majority of patients with an eating disorder had renal impairment. PdCr was more sensitive in the detection of renal impairment in individual patients compared to eGFR.

## 1. Introduction

Impairment of kidney function can be detected by relating the blood levels of creatinine, a substance produced by muscle tissue and passing freely through the kidney into urine, to an age-related reference range. An elevation of creatinine above this normal range indicates an inability of the kidneys to let this substance pass into urine, indicating impairment of kidney function. People who are taller have, on average, a greater muscle mass, and it is, therefore, required to adjust the creatinine level for body height using a formula (see method section below), arriving at an estimated glomerular filtration rate (eGFR). A retrospective analysis of the estimated glomerular filtration rate (eGFR) results of 111 patients in hospital with a severe eating disorder found impaired renal function in 33% [1]. Another study of hospitalized adolescent patients with severe malnutrition found 72% had impaired kidney function, including 59% with mild impairment as evident from an eGFR of between 89 and 60 mL/min, 12% with moderate impairment (eGFR between 59 and 45 mL/min), and 2% with severe impairment (eGFR between 44 and 30 mL/min) [2]. Risk factors for renal impairment identified in those studies included the following: low body mass index (BMI), dehydration, rhabdomyolysis, nephrocalcinosis, bradycardia, hypophosphatemia, and hypokalemia [3]. According to a further study, patients (predominantly adults) with anorexia nervosa (n = 2091) had, compared to controls, a significantly higher risk of acute dialysis (adjusted hazard ratio 2.10 [95% confidence interval 1.19–3.68]), nephritis, acute renal failure, and chronic renal failure [4]. This highlights the importance of early detection of renal impairment in patients with eating disorders to prevent severe and chronic renal failure. Dehydration was suggested as one of the factors causing renal impairment in patients with eating disorders. The patients are trying to reduce body size and decrease weight via fluid restriction. As a consequence, the body tries to preserve water through the release of a hormone called vasopressin or antidiuretic hormone (ADH) in response to a reduced cellular volume in dehydration and reduced pressure inside the “antechambers” (atria) of the heart. ADH release is accompanied by a release of a substance called C-terminal preprovasopressin (co-peptin), which is more stable than ADH and, therefore, reflects the water balance in the body over a longer term. In a large prospective study in Sweden (Malmoe diet and cancer cardiovascular cohort), copeptin and eGFR were measured at baseline and, after a follow-up time of about 17 years, in 3186 patients. After multivariate adjustment (gender, age, baseline eGFR, smoking status, systolic blood pressure, antihypertensive treatment, and follow-up time), copeptin was independently associated with the deterioration of eGFR. Each increase in copeptin by one standard deviation independently predicted chronic kidney disease occurrence [5]. In an epidemiological study conducted in Australia at a specified point in time, total fluid intake was assessed in 5220 adults. Subjects with the highest water consumption (3.2 L/d) had a significantly reduced risk of chronic kidney disease [6]. The analysis of data from the National Health and Nutrition Examination Survey in the United States, including 3427 adults, of whom 13% had CKD, revealed that CKD was significantly more common in those with the lowest total water intake [7].

Thirst here does not prevent self-imposed low-grade dehydration: Thirst is triggered by a concentration of solutes in the blood, which is above that associated with a release of ADH. ADH secretion begins at about 280 mosm/L. For the feeling of thirst to be noticed, plasma osmolality has to reach 290 mosm/L. Therefore, thirst is a poor indicator of significant low-grade dehydration, leading to increased ADH levels [8].

Higher ADH levels lead to an increase in blood flow within the kidneys and the filtration rate of urine. This was demonstrated by giving ADH to rats, and it increased blood flow and urine filtration rate by 40% [9]. Greater water intake was furthermore associated with increased urine filtration caused by a protein meal, but this was reduced in subjects with better hydration [10]. Stimulation of the receptor for ADH was associated with five-fold increased albuminuria, increased mortality rate, and greater cardiac and renal hypertrophy (hypertrophy is derived from ancient Greek meaning overgrowth) compared to controls. Microscopically visible kidney abnormalities were made worse, and this effect could be prevented by substances blocking this stimulation [11].

These studies used creatinine for the calculation of eGFR, which served to define the reduction in renal impairment. Creatinine is derived from muscle or dietary intake of muscle protein and will, therefore, overestimate GFR in patients with an eating disorder due to their low muscle mass and low protein intake [12], similar to patients with neurodisability-related muscle wasting [13]. In the latter, we found that children in whom walking ability is severely limited, who use a wheelchair most of the time, or who have more severe impairment in all areas of motor function, making them unable to sit or stand independently and resulting in severe muscle wasting, had a creatinine level when well with a mean of 30.7 micromol/L (n = 65) compared to normal controls with a mean of 36.5 micromol/L at an age of around 6 years on average. In this group with severe muscle wasting, creatinine levels were with a mean of 25.7 micromol/L, significantly lower in patients who died compared to survivors with 31.1 micromol/L. This highlighted that muscle wasting, as reflected in creatinine levels, is also related to mortality. This means that, particularly in patients with the most severe forms of eating disorder, i.e., those who are most severely affected by muscle wasting, including myocardium, the risk of not recognizing renal failure due to dehydration upon admission is highest. Creatinine within the normal range, according to the laboratory reference, would, in those patients, indicate significant renal impairment. Myocardial weakness, together with dehydration, may put the patient at risk of collapse due to loss of consciousness from hypovolemia-related hypotension. Other markers, like cystatin C and radionuclide-based determination of GFR, which are not significantly affected by muscle mass or muscle protein intake, were found not to accurately reflect GFR in patients with eating disorders [14,15]. It is, therefore, pivotal to enable personalized medicine to find a tool which can detect renal impairment in the individual patient, which is not reliant on reference ranges obtained in patients without muscle wasting and becomes, like a single measurement of creatinine, inaccurate as dependence on muscle mass is not taken into account. Such a tool needs to be readily applicable by the clinician at the bedside without specific training, without drawing on additional laboratory resources, or without involving the opinion of a nephrologist. A more accurate and readily applicable tool for the detection of dehydration-related renal impairment enables the targeting of fluid intake for the individual patient, thus fulfilling the goals of personalized or precision medicine.

### Objectives

Our objective was to investigate the percentage drop in creatinine during nutritional rehabilitation and rehydration as a new indicator of renal dysfunction that is not dependent on muscle mass for assessing renal dysfunction in patients with eating disorders.

We also aimed to compare the results of creatinine and height-based GFR estimation on admission with a percentage drop in creatinine as a marker of renal dysfunction.

Finally, we aimed to investigate the cause of renal dysfunction as evident from the percentage drop in creatinine by correlation of this drop with Z-score centile of body weight, heart rate on admission, systolic and diastolic blood pressure centile, and haematocrit and sodium level upon admission.

## 2. Methods

In this retrospective cohort study, we investigated renal function in all children admitted consecutively because of an eating disorder to a paediatric unit during a 5-year period. The classification of the eating disorder followed the *DSM-5*. All patients admitted were put on a standardized refeeding program starting with 1200 kcal/day and subsequent increments of calories to enable a weight gain of at least 0.5 kg/week with no variations of protein intake per kilogram body weight per day between patients. This project was certified by the Health Research Authority of the United Kingdom as not requiring approval by a local research ethics committee as it was an audit project for assessment of service provision for patients with eating disorders admitted to hospital [16]. The project was registered as audit project with the Clinical Quality and Governance Department of the Luton & Dunstable University Hospital. Patient consent was not required due to anonymous retrospective case record review for audit purposes. Renal function was expressed as eGFR and the novel marker percentage drop in creatinine. Renal function was expressed as eGFR using the modified bedside Schwartz method: estimated glomerular filtration rate (mL/minute/1.73 m^2^) = 35 × height (cm)/serum creatinine (micromol/litre) from creatinine on admission [17]. Percentage drop in creatinine was calculated from the percentage drop of creatinine from admission to the lowest creatinine on daily monitoring. Daily monitoring was for all patients set for one week by the lead clinician for eating disorders as local protocol to detect any features of refeeding syndrome. It included measurement of sodium, potassium, urea, creatinine, phosphate, magnesium, alanine aminotransferase, haemoglobin, haematocrit, white cell count, platelet count, and blood gas analysis. The blood samples were analysed on-site in the admitting hospital in the laboratories for clinical biochemistry, and the method used for measurement of creatinine was the enzymatic method on the Roche C702 module on the Cobas 8000 analyser (Roche Diagnostics International Ltd., CH-6343 Rotkreuz Switzerland). In patients where there were further creatinine measurements available from follow-up after the ward admission, these were included in the analysis. Data on laboratory parameters were extracted anonymized from a hospital computer database. Individual patient data relating to patient characteristics were extracted by both investigators independently from electronic records of each patient retrospectively. Data were recorded in an anonymized way on Excel (Microsoft Windows) spreadsheets on password-protected hospital computers in the hospital both authors were employed. The percentage drop in creatinine was correlated with eGFR and physical parameters, including Z-score centile for body weight (using a calculator based on Centre for Disease Control, Atlanta, USA data (https://peditools.org/growthpedi/; last accessed on 28 May 2024), heart rate, systolic and diastolic blood pressure centile, and postural drop in blood pressure. We compared percentage drop in creatinine in patients with and without symptoms of cardiovascular instability, like fainting and dizziness. If the percentage drop in creatinine was related to dehydration, more intensive fluid management should lead to a greater drop in creatinine. We, therefore, compared the drop in creatinine in patients with and without fluid management plan upon admission. In patients with a fluid management plan upon admission, we planned to administer 45 mL/kg/day (SD 8.5) of oral or intravenous fluids. We regarded an association of percentage drop of creatinine with active rehydration as confirmation of the percentage drop in creatinine being due to resolution of dehydration-induced renal impairment.

The severity of renal impairment was classified following the National Kidney Foundation’s Kidney Disease Outcomes Quality Initiative clinical practice guidelines for chronic kidney disease in children and adolescents (See Table 1) [18].

### Statistical Analysis

Because of the right-skewed distribution of data for creatinine change during admission and body weight Z-score centiles, we used non-parametric data analyses, including medians and Mann–Whitney U test for comparison of continuous data and Spearman’s rho for correlation analyses. *p*-values were reported in their two-tailed version, and a *p*-value of less than 0.05 was taken as indicative of a statistically significant low probability of an erroneous rejection of the null hypothesis. The statistical software used was SPSS version 29 (IBM).

## 3. Results

We included 29 patients, out of which 27 were female and 23 had a diagnosis of anorexia nervosa, 5 had unspecified feeding or eating disorder, and 1 had avoidant/restrictive food intake disorder. The mean age was 13.4 years (range 4 to 16 years), and the admission Z-score centile for bodyweight for age had a median of 36.7 (range 0.1 to 94.6). BMI on admission had a median of 17.4 (range 12.3 to 26.6), and BMI centiles (UK and WHO data sets underlying the BMI centile charts) had a median of 25 (range <0.01 to 91). Duration of illness, where recorded before admission, had a median of 6.0 months (range 0.25 months to 60 months), and weight loss, where recorded before admission, had a median of 8.2 kg (range 0–30 kg). All patients were admitted to the hospital for nutritional rehabilitation as this could not be provided at home. The lowest creatinine level was used to calculate the percentage drop in creatinine compared to that upon admission, and the highest estimated GFR was identified at a mean of 5.7 days (range 2 to 17) after admission.

A percentage drop in creatinine after admission was noted in 26/29 (89.7%). The eGFR, which could be calculated in 23 patients upon admission, was <90 in 15 (65%) and improved to >90 in 13/15 (86.6%) during hospital treatment. The severity of renal impairment was mild in 14 and moderate in 1 patient. In the patients where the eGFR did not normalize, it improved in one patient from 63 to 85 during the admission and was normal at 94 upon follow-up. In the other patient, it improved from 77 to 89 during the admission, and follow-up data were unavailable as the patient was subsequently cared for at another hospital.

**Figure 1:**
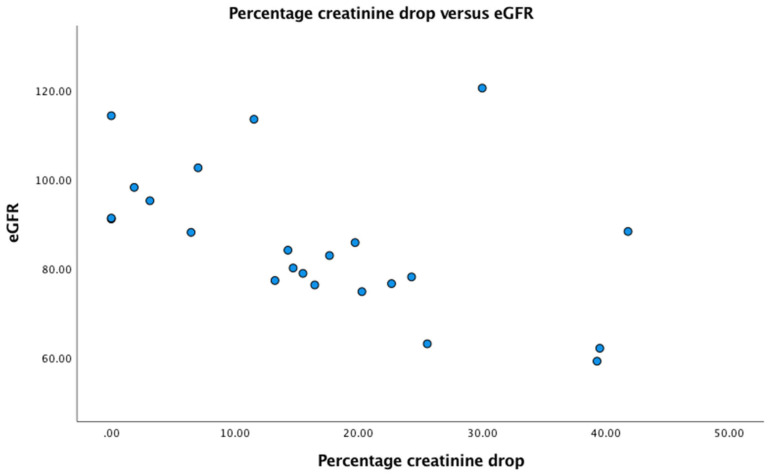
Scatter plot of eGFR in mL/min/1.73 m^2^ versus percentage drop in creatinine (%).

In patients with a normal eGFR of >90 upon admission (n = 9), three (33%) had no drop in creatinine on subsequent measurements during nutritional rehabilitation, and 6 had a drop in creatinine of 1.8, 3.1, 7.0, 11,5, 14.2, and 30.0%, respectively. All patients with a reduced GFR on admission also had a drop in creatinine after admission. In Figure 1, we plotted eGFR versus percentage drop in creatinine. There was a highly significant negative correlation between the percentage drop in creatinine and eGFR (r = −0.58, *p* = 0.003).

Two of the patients had hypokalemia upon admission (6.9%): one of 1.8 and the other of 1.9 mmol/L, respectively, with the latter also being the only patient with low phosphate of 0.68 mmol/L upon admission. In both patients, the low potassium was due to vomiting-induced metabolic alkalosis, and the eGFR upon admission was 80 and 88, respectively, and normalized upon intravenous fluid management. None of the patients had hyponatremia or features of rhabdomyolysis.

### 3.1. Percentage Drop in Creatinine, Laboratory Parameters Which May Be Altered in Dehydration, and Relation to Fluid Management

To investigate whether the percentage drop in creatinine was related to admission haematocrit level or sodium levels, which are parameters with possible elevation in dehydration due to a restriction in water intake, we performed correlation analyses. Neither parameter was significantly correlated with a percentage drop in creatinine with haematocrit (correlation coefficient −0.24, *p* = 0.21) and sodium (correlation coefficient −0.26, *p* = 0.16).

**Figure 2:**
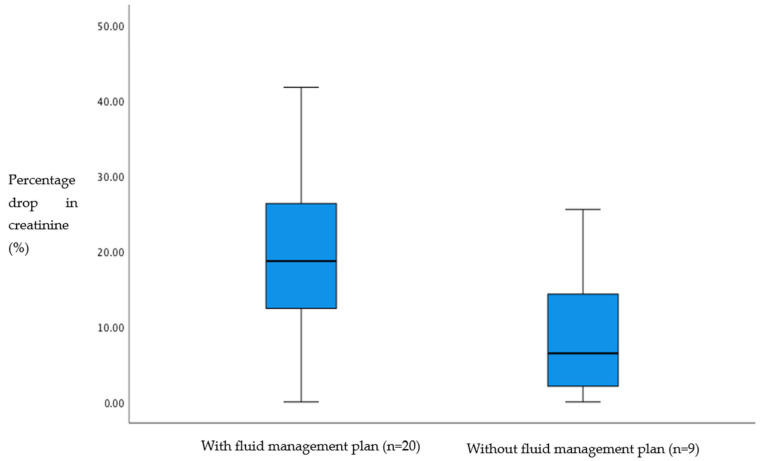
Percentage drop in creatinine and presence of fluid management plan upon admission (bar inside the box = median, box = interquartile range, whiskers = outliers).

In patients with a fluid management plan upon admission (n = 20), the percentage of decreased creatinine had a median of 18.6%. Without such a plan (n = 8), it was 6.4% (*p* = 0.02) (see Figure 2).

### 3.2. Percentage Drop in Creatinine and Features of Cardiovascular Compromise upon Admission

We compared the percentage drop in creatinine in patients with and without a history of fainting and/or dizzy spells before admission and found no difference (median of 18.6% with (range 4 to 39) versus 15.6% without (range 2 to 41)).

We performed a correlation analysis of heart rate, systolic and diastolic blood pressure centiles, and systolic or diastolic blood pressure drop on orthostasis with creatinine drop, and there was no significant correlation of those cardiovascular parameters with creatinine drop after admission (all *p* > 0.2). eGFR was also not significantly correlated to heart rate upon admission.

There was a significant correlation between systolic (r = 0.69, *p* < 0.001) and diastolic (r = 0.60, *p* = 0.003) blood pressure centiles and heart rate upon admission.

### 3.3. Nutritional Status and Parameters of Renal Dysfunction upon Admission

There was no significant correlation between body weight Z-score percentile on admission and creatinine drop: r = −0.09, *p* = 0.64. eGFR was equally not correlated with body weight Z-score percentile: r = 0.26, *p* = 0.22.

## 4. Discussion

Our study demonstrated that the percentage drop in creatinine during nutritional rehabilitation is a parameter of renal dysfunction more sensitive than eGFR. An apparently normal eGFR does not rule out significant renal dysfunction in this population of underweight children with an eating disorder because of the reduced muscle mass leading to an overestimation of eGFR (see introduction). The percentage drop was most likely due to rehydration, as fluid management was significantly associated with this drop in creatinine. There was no evidence to support alternative explanations like nutritional status and cardiovascular compromise as causes of the renal impairment observed. The findings are, therefore, consistent with the percentage drop in creatinine reflecting dehydration-induced renal impairment upon admission. Using the percentage creatinine drop for detection of dehydration-induced renal impairment detected more children with significant dehydration. This approach, therefore, enables more personalized fluid management in patients in the long term. The lack of correlation in the percentage drop in creatinine with haematocrit values ruled out the influence of the haemo-concentration of the initial blood sample and supports the presence of genuine renal dysfunction in patients with a drop in creatinine.

The lack of a correlation of cardiovascular parameters with the renal dysfunction we detected suggests that dehydration was not a cause of cardiovascular compromise. Bradycardia-associated reduced cardiac output appeared to be the cause of an observed lowering of blood pressure. Our findings are in keeping with a recent retrospective study which found no difference in the BMI of patients with abnormally low eGFR and is in contrast to the finding that there was a significant positive correlation between heart rate upon admission and eGFR [19].

In a previous retrospective study, it was noted that among those with admission eGFR < 90, in 20/37 (54%), this improved to 90 mL/minute/1.73 m^2^ or more prior to discharge [1]. Our result of a greater percentage of 86.6% of patients with normalization of GFR may indicate that our children, who were likely, on average, younger, had a shorter duration of physical impairment, including dehydration, leading to a more rapid resolution of renal impairment. Otherwise, our proactive fluid management strategy, provided for the majority (69%), enabled a more rapid and complete restoration of renal function. Future research can use the percentage drop in creatinine as an outcome measure of interventions upon admission to improve fluid intake. To obtain a better impression of the acute status of hydration, one could investigate co-peptin and urine osmolality upon admission as a very short-term measure of hydration status and relate this to the percentage drop in creatinine.

Future research needs to further investigate the renal function of patients with eating disorders from the onset over the years into late adulthood in the long term in a longitudinal, multicentre, multinational prospective study to further identify risk factors for future severe and chronic renal impairment in relationship to daily fluid intake and measures of dehydration like urine osmolality [20,21,22,23]. Such an investigation needs to be complemented by an analysis of a detailed fluid intake diary in prospective studies. Such a diary should become part of the routine long-term care of patients with eating disorders.

Shortcomings of using percentage creatinine drop as an assessment of dehydration-induced renal impairment include that as opposed to eGFR, which could, in our study, even with reduced muscle mass in our population, detect renal impairment in the majority using a single creatinine measurement upon admission, the percentage drop in creatinine required repeat measurement days later. The percentage drop in creatinine may, in addition, still have underestimated the degree of renal impairment as a rehydration-induced reduction in creatinine may have been lesser in patients with significantly increased muscle protein intake provided during nutritional rehabilitation. Increased muscle protein intake means increased creatinine intake, which can increase serum creatinine levels [24].

The medical records analysed did not allow for the assessment of any influence of hydrating and dehydrating food or fluid items. It is important to compensate with water consumption for the effect of ingredients in drinks and food, which contain substances that have so-called diuretic effects or cause water loss through diarrhea. Diuretic effects mean they cause increased water loss through the kidneys, which is noticeable via the need to pass water more often than before and in larger volumes. Caffeine is contained in a number of soft drinks consumed by children and present in all chocolate other than white chocolate with the concentration increasing the darker it is. In an investigation including 12 adults with no medical conditions and a mean age of 24 years, participants were subjected to exercise in a 35 °C, 61% relative humidity environmental chamber and could drink 2 L of a caffeinated drink, giving on average 4 mg/kg of caffeine or water (control group). The group receiving the caffeinated drink had a significantly greater reduction in kidney function, as evident from changes in serum creatinine. The fact that this was due to dehydration is supported by the fact that serum co-peptin levels rose significantly more in the group taking caffeinated drinks [25]. Strong evidence for an increase in urine output with high doses of caffeine is derived from prospective observational studies using 8.2 to 10.2 mg/kg/day of caffeine equivalent to consumption of three to four cups of coffee per day and demonstrated an increase in urine output by more than 40% per day together with a reduction in body weight and water content [26].

Future studies need to register and quantify caffeine-containing food being consumed during nutritional rehabilitation and rehydration and investigate its impact on recovery of renal function.

The percentage drop in creatinine can guide long-term management via the identification of otherwise undetected fluid restriction as part of the pathology of the eating disorder of the individual patient. This will help in communication with the patient and highlights the importance of focusing on individually adequate fluid intake, which, for all children in this study, would have been at least 2 L per day [27]. It is known that if water intake is reduced below requirements that via an induction of vasopressin release, the release of the adrenocorticotropic hormone is stimulated, and, therefore, cortisol with its psychotropic effects, which include increased anxiety and low mood. In a trial that used the subjects as their own control group, they were first exposed to reduced fluid intake with an assessment of mood afterwards followed by re-hydration with a defined amount of water and the mood re-assessed: The 12 men from a university in China (Cangzhou) had after 12 h of no fluid intake during night tests at baseline. The concentration (in the form of osmolality) of blood and urine was analysed to measure the state of hydration. The investigation then employed a profile of mood states questionnaire. The participants had to apply the questionnaire to themselves and estimate their emotional condition using seven subscales with a total of 40 adjectives that assessed for total mood disturbance (TMD): tiredness, depression, tension, strength, anger, confusion, and esteem-related affect. The subjects were afterwards asked not to consume water for 36 h but could eat on day 3. On day 4, the assessments were repeated. At half past eight in the morning, trial subjects were allowed to have 1500 mL of water. After one hour, the assessments were repeated again. In comparison with assessment results at baseline, participants had significantly lower scores of esteem-related affect and vigor. Rehydration resulted in a reduction in fatigue and mood disturbance. Increased water intake can, therefore, reduce anxiety and low mood [28]. The percentage drop in creatinine may, therefore, guide clinicians in estimating the contribution of dehydration to the current mental health of the patient affected by an eating disorder and help with emphasis in the communication to carers regarding monitoring and encouraging adequate water intake in the individual patient.

## 5. Conclusions

The majority of patients with an eating disorder had renal impairment. PdCr was more sensitive in the detection of renal impairment compared to eGFR.

## Figures and Tables

**Table 1:** Classification of stages of chronic kidney disease (National Kidney Foundation’s Kidney Disease Outcomes Quality Initiative).

Stage	GFR (mL/min/1.73 m^2^)	Description
1	≥90	Kidney damage with normal or increased GFR
2	60–89	Kidney damage with mild reduction in GFR
3	30–59	Moderate reduction in GFR
4	15–29	Severe reduction in GFR
5	<15 or dialysis	Kidney failure

## Data Availability

The data that support the findings of this study are available upon request from the corresponding author. The data are not publicly available due to privacy or ethical restrictions.
